# Do we need a health technology assessment approach for non-inferiority specifically? A comment on: clinical equivalence and non-inferiority within health technology assessment by Taylor et al. (2025)

**DOI:** 10.1007/s10198-025-01840-0

**Published:** 2025-09-25

**Authors:** Beryl Primrose Gladstone, Werner Vach

**Affiliations:** 1 Infectious diseases Division, Department of Internal Medicine I, University Hospital Tübingen, Tübingen, Germany; 2 DZIF-Clinical research unit, Infectious diseases, Internal Medicine I, University Hospital Tübingen, Tübingen, Germany; 3 Basel Academy for Quality and Research in Medicine, Basel, Switzerland; 4 Department of Sport Sciences and Clinical Biomechanics, University of Southern Denmark, Odense, Denmark

**Keywords:** Health technology assessment, Non-inferiority, Non-inferiority margin, Advantage deficit assessment

## Abstract

Taylor and colleagues presented a very informative and comprehensive overview on the current practice of performing non-inferiority analysis in HTA. We would like to point out that HTA of potentially non-inferior technologies should be based on balancing potential advantages against potential deficits exactly as for potentially superior technologies.

New health technologies are often motivated by being potentially superior to existing ones with respect to patient relevant outcomes. However, sometimes their advantage refers to other dimensions such as less side effects or lower costs, whereas they only claim to be non-inferior with respect to patient relevant outcomes. Taylor and colleagues [1] presented a very informative and detailed account about the current practice of performing HTA in the latter setting.

We highly appreciate the research work which gives a comprehensive overview and addresses many important issues arising in this context. As seen in the drug regulatory approaches in practice, Taylor et al assume that HTA of potentially non-inferior technologies require a different approach than HTA of potentially superior technologies. It is of interest to note, that none of the HTA and regulatory bodies reviewed in the paper nor any of the stakeholders interviewed seem to have questioned this assumption. However, we believe that it is worth to question this assumption, as it may allow a different perspective at the problem.

The core of HTA is to identify and quantify the potential advantages and deficits of a new technology in order to support a decision making by different stakeholders. HTA provides many techniques to support stakeholders in balancing between advantages and deficits, e.g. by considering specific ratios or visualizing the available information in advantage-deficit planes.

For potentially superior technologies, the potential advantage is an improvement in patient relevant outcomes, whereas potential deficits may be related to harms, safety risks, usage burden, costs, ethics or societal impact. Technically speaking, the concept is the same for potentially non-inferior technologies, just that it is the other way around. Here, advantages are expected with respect to harms, safety, costs, usage burden or societal impact, and a potential deficit may be with respect to patient relevant outcomes. Hence, we do not require new techniques to balance advantages and deficits, as it is only the direction of interpretation which changes. For example, instead of asking "Will a patient accept the risk increase for a specific adverse event by a factor of y if the expected outcome is increased by a factor x?”, the question is now “Will a patient accept a decrease in an expected outcome by a factory y if the risk for a specific adverse event decreases by a factor x?”.

It may be argued that, in non-inferiority settings, the advantage is often of implicit nature and hence impossible or difficult to quantify. Indeed, a corresponding empirical investigation [2] revealed that the vast majority of NI trials does not systematically collect data to support the expected advantages. However, it was also found that half the NI trials collect data for at least for one of the potential advantages and a more systematic collection of these data would have been feasible in the majority of NItrials. Only 19 out of 170 advantages claimed in 87 non-inferiority trials could be regarded as non-assessable because they referred to advantages at the health care system level.

This is, however, not surprising considering the perspective mentioned above: HTA of potentially non-inferior technologies requires data of the same dimensions as HTA of potentially superior technologies. In both the situations, RCTs are typically to provide these data and there is no reason to believe that there are fundamental differences in the potential to collect these data. The lack of data on potential advantages in non-inferiority trials, most probably, reflects their poor quality, which was pointed out by Taylor et al [1] as well. It is important to note that balancing between advantages and deficits by stakeholders does not require to agree on a non-inferiority margin. It rather requires an agreement on thresholds for certain ratios, just as it may be the case for any HTA.

Based on these considerations, we believe that it is worthwhile to take the advantage-deficit perspective into account when considering HTA of potentially non-inferior technologies. It provides an approach which avoids in-depth discussions about non-inferior margins. We do not claim that this would solve all NI related analytical issues, however it provides a useful addition or alternative. Consequently, we suggest to extend the fourteen “recommendations for assessing claims of non-inferiority or clinical equivalence” presented in Table 4 of Taylor et al[1] by a further recommendation:

- HTA of potentially non-inferior technologies should be based on balancing potential advantages against potential deficits exactly as for potentially superior technologies. Non-inferiority trials are hence encouraged to collect data on all potential advantages and disadvantages applying the same standards and rigour as in superiority trials and these data should inform the HTA.
